# Functional Biomechanical Tests of the Foot and Ankle in Physiotherapy and Sports—Outcome Measures, Wearable Sensor Integration, and Psychometric Properties: A Systematic Review

**DOI:** 10.3390/jcm15103892

**Published:** 2026-05-18

**Authors:** Guna Semjonova, Rodrigo Vallejo-Martínez, Luis Ceballos-Laita, Sandra Jiménez-del-Barrio, Sergejs Davidovics, Anna Davidovica

**Affiliations:** 1 Department of Rehabilitation, Riga Stradins University, 16 Dzirciema Street, LV-1007 Riga, Latvia; guna.semjonova@rsu.lv (G.S.);; 2 Grupo de Investigación Clínica en Ciencias de la Salud, Facultad de Ciencias de la Salud, Universidad de Valladolid, 42004 Soria, Spain

**Keywords:** foot, ankle, wearable sensors, gait, psychometrics, validity, reliability, SEM, MDC, MCID, sports biomechanics

## Abstract

**Objectives**: To systematically synthesize existing evidence on functional biomechanical tests of the foot and ankle in physiotherapy and sports, focusing on their outcome measures, compatibility with wearable sensor technologies, and psychometric properties. **Methods**: We performed a systematic review (PRISMA-guided) of PubMed, Web of Science, PEDro, and SPORTDiscus from inception to December 2025. Eligible studies evaluated functional foot/ankle biomechanics in athletes, healthy adults, or adults with musculoskeletal foot/ankle conditions using wearable sensors (e.g., IMUs, wireless pressure insoles). Two reviewers independently screened, extracted data, and appraised methodological quality using the COSMIN Risk of Bias tool, applying property-specific ratings. Heterogeneity precluded meta-analysis; findings were narratively synthesized and tabulated. **Results**: Twenty full texts were reviewed; four studies (*n* = 83 participants) met the inclusion criteria. Wearable devices included foot- or trunk-mounted IMUs and wireless pressure insoles. Reported outcomes spanned temporal gait events and inner-stance phases, vertical ground reaction force (vGRF) and centre-of-pressure trajectories, running step rate/stride length, and jump counts in competition. Validity was most frequently assessed: foot-worn IMUs showed millisecond-level agreement with in-shoe pressure references for stance and inner-stance events; pressure insoles demonstrated acceptable agreement with force plates for vGRF/COP alongside fair-to-excellent test–retest reliability; foot- vs. shank-mounted IMUs provided strong agreement for running step rate and stride length; and competition-based jump detection using IMUs achieved high sensitivity. Across studies, reliability indices were inconsistently reported, measurement error (SEM/MDC) was sparse, and MCID was not reported. The COSMIN appraisal ranged from very good/adequate to inadequate, driven primarily by small sample sizes, non-gold-standard comparators, and incomplete psychometric reporting.

## 1. Introduction

Foot and ankle injuries represent a significant burden in both athletic and general populations. Lateral ankle sprains are among the most common musculoskeletal injuries in sport, alongside overuse conditions such as Achilles tendinopathy and plantar fasciopathy [1,2]. Surveillance systems, including the National Collegiate Athletic Association [3], confirm their high incidence, while degenerative and post-surgical conditions, such as ankle osteoarthritis and total ankle replacement, further contribute to gait impairment and reduced quality of life [4].

Given the central role of the foot and ankle in load transfer, balance, and propulsion, functional biomechanical assessment is essential for rehabilitation and return-to-sport decision-making. Tests such as gait analysis, hop-and-jump tasks, balance assessments, and sit-to-stand tasks are commonly used to evaluate mobility, neuromuscular control, and mechanical loading patterns [5,6]. Outcome measures, including temporospatial parameters, ground reaction forces, and centre-of-pressure trajectories, provide insight into asymmetries and residual deficits following injury.

However, traditional assessments remain limited. Clinical evaluation often relies on visual observation and subjective scoring, which are prone to inter-rater variability and low sensitivity to subtle biomechanical alterations [7]. Even instrumented laboratory systems, such as motion capture systems or force plates, are constrained by cost, limited portability, and the need for controlled environments.

Wearable sensor technologies have emerged as tools to extend biomechanical assessment beyond the laboratory. Inertial measurement units (IMUs), plantar pressure insoles, smart textiles, and surface electromyography (EMG) systems allow continuous data acquisition during walking, running, jumping, and sport-specific activities [8]. Validation studies report agreement between wearable-derived metrics and gold-standard systems; IMUs show good concurrent validity for temporospatial gait parameters compared with motion capture [9], and instrumented insoles demonstrate acceptable agreement with force plates for vertical ground reaction forces [10]. Moreover, wearable devices have been implemented in competitive sport settings to quantify mechanical load in real time.

Despite these advances, relevant gaps persist. There is marked heterogeneity in functional tests, sensor placement, and validation protocols, hindering comparison across studies. While concurrent validity is frequently examined, other psychometric properties, such as test–retest reliability, measurement error, minimal detectable change (MDC), and minimal clinically important difference (MCID), are inconsistently reported, limiting clinical interpretability.

Therefore, a structured synthesis is needed to clarify which functional biomechanical tests of the foot and ankle have been evaluated using wearable technologies, which outcomes have been reported, and whether their psychometric properties support clinical and performance applications. Accordingly, this systematic review aimed to synthesize the available evidence on functional biomechanical tests of the foot and ankle used in physiotherapy and sports contexts, with a focus on reported outcome measures, compatibility with wearable sensor technologies, and psychometric properties. Additionally, the review sought to identify gaps and future opportunities for the integration of artificial intelligence (AI) and digital health approaches in foot and ankle biomechanical assessment.

## 2. Materials and Methods

### 2.1. Study Design

This systematic review of the literature was performed following the PRISMA statement and the Cochrane recommendations [11] (Supplementary Materials). The protocol was registered prospectively in PROSPERO with a unique identification number (ID: CRD420251155532) [12].

### 2.2. Search Strategy

Bibliographical searches were carried out in PubMed (MEDLINE), Web of Science (WoS), Physiotherapy Evidence Database (PEDro), and SportDiscus from inception to December 2025. Medical Subject Headings (MeSH) terms combined with grey terms were used as keywords in the search strategy. The terms used and the search strategies used in each database are described in detail in Appendix A. The reference lists of the included studies and the above-mentioned previous studies were also hand-searched.

### 2.3. Eligibility Criteria and Study Selection

Studies were included if they involved athletes of any competitive level (from amateur to professional), adults with musculoskeletal foot and/or ankle injuries, or healthy participants undergoing functional biomechanical assessment of the foot and ankle. Eligible study designs comprised observational studies (cross-sectional, cohort, and case–control), and psychometric or measurement property studies (including reliability, validity, and responsiveness of functional biomechanical tests) evaluating functional foot and ankle biomechanics using wearable sensor technologies. Studies were excluded if they included participants with neurological disorders affecting gait or balance (e.g., stroke, Parkinson’s disease, or multiple sclerosis), systemic conditions not primarily related to musculoskeletal foot or ankle health (e.g., diabetes-related neuropathy or rheumatoid arthritis), or populations younger than 18 years or older than 70 years. Systematic reviews, meta-analyses, narrative reviews, case reports, case series, and expert opinion articles were excluded.

The reference lists retrieved from each database were exported to Mendeley to remove duplicates. Two authors (L.C.-L. and R.V.-M.) independently reviewed the titles and abstracts of each retrieved study to determine their eligibility. The studies that met the eligibility criteria were assessed in full text by the same authors. A third author (G.S.) was consulted in case of discrepancies.

### 2.4. Data Extraction

Data extraction was conducted independently by two reviewers (L.C.-L. and G.S.) using a predetermined data extraction form adapted from the Cochrane Collaboration. The extraction form included information on study population, device used, outcome measures (functional tests), and psychometric properties, including test–retest reliability, intra-rater and inter-rater reliability, validity, standard error of measurement (SEM), minimum detectable change (MDC), and minimum clinically important difference (MCID); however, only the variables reported in each study were extracted and synthesized.

### 2.5. Risk of Bias and Methodological Quality Assessment

The methodological quality and risk of bias of the included studies were assessed using the COSMIN (Consensus-based Standards for the selection of health Measurement Instruments) Risk of Bias tool [13]. COSMIN is specifically designed to evaluate studies investigating the measurement properties of instruments, such as criterion validity, reliability, and measurement error.

Unlike generic quality assessment tools, COSMIN does not apply a fixed set of items to all studies. Instead, only the items relevant to the specific measurement property under investigation are evaluated. Consequently, not all COSMIN items were applicable to every included study. For example, studies assessing criterion validity were evaluated on items related to study design, adequacy of the reference standard, sample size, and statistical methods. In contrast, items related to test–retest reliability or measurement error were not assessed when these properties were not part of the study objectives.

This property-based approach is consistent with COSMIN methodological guidelines and prevents penalizing studies for not evaluating measurement properties that they did not intend to assess. For each measurement property, the overall risk of bias rating was determined according to the COSMIN principle, which states that the lowest-rated item determines the overall judgement.

### 2.6. Data Synthesis and Analysis

Data from the included studies will be synthesized using a structured narrative approach and summarized in tabular form. Given the anticipated heterogeneity in study designs, participant characteristics, sensor types, outcome measures, reference standards, and validation protocols, a quantitative meta-analysis will not be conducted. For each included study, extracted data will include information on study design, population characteristics, measurement conditions, index instruments, reference standards, statistical methods, and reported outcomes related to measurement properties. Studies will be grouped according to the measurement properties assessed, such as criterion validity, reliability, and measurement error. The synthesis will integrate both the reported results of measurement property evaluations and the methodological quality of the contributing studies.

## 3. Results

**Figure 1:**
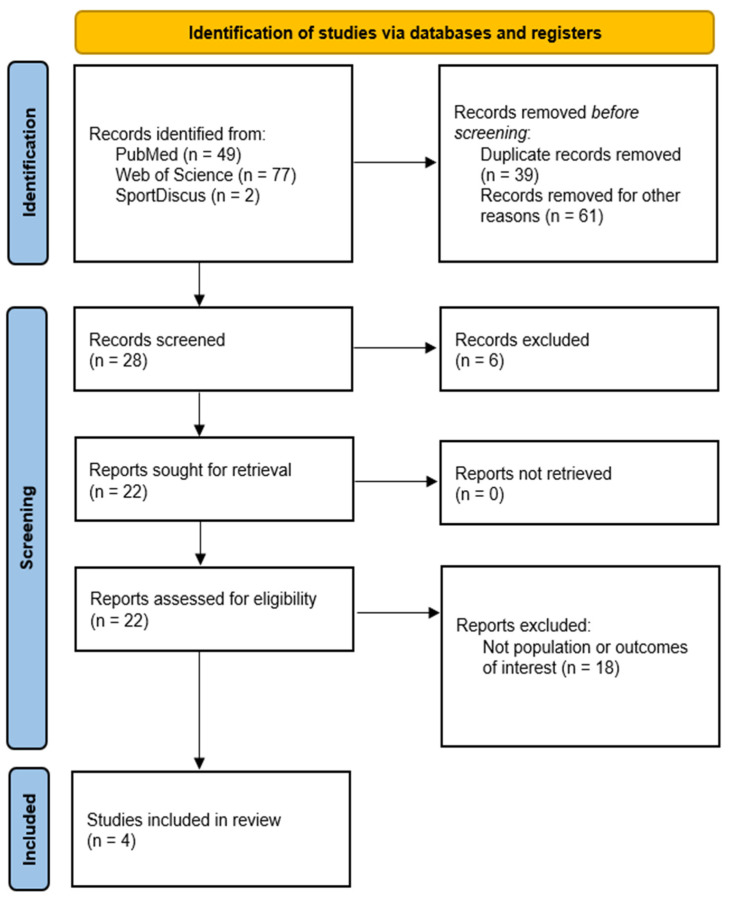
Flowchart diagram of the included studies.

Our searches yielded 128 records. After removing 39 duplicates, 89 records remained, of which 61 were excluded based on title screening because the populations did not meet the eligibility criteria or the study designs were not aligned with the predefined inclusion criteria. Subsequently, 28 records underwent title and abstract screening, and 20 studies were selected for full-text review. Finally, four studies met the inclusion criteria and were included in this systematic review (Figure 1).

### 3.1. Characteristics of the Included Studies

Four studies were included in this systematic review, comprising a total of 83 participants across healthy adults, athletes, and individuals with musculoskeletal foot and ankle conditions. The included studies were published between 2013 and 2025 and primarily employed observational or experimental non-randomized designs focused on functional biomechanical assessment using wearable sensor technologies. One study investigated adults with ankle osteoarthritis, as well as post-surgical patients following total ankle replacement and ankle arthrodesis, alongside healthy controls, during overground walking using foot-worn inertial sensors validated against pressure insoles [14]. Two studies exclusively involved healthy or injury-free adults, evaluating functional tasks such as walking, running, squats, jumps, sit-to-stand, and stair negotiation using either inertial measurement units or wireless pressure insoles [15,16]. One study focused on elite beach volleyball players, assessing jump detection during official competition using wearable inertial sensors validated against video-based analysis [17]. Across all included studies, wearable devices such as foot-worn or body-mounted inertial measurement units and pressure insoles were used to capture functional biomechanical outcomes. The primary outcome measures included temporal gait parameters, ground reaction force variables, centre-of-pressure trajectories, and jump detection metrics. A summary of the study characteristics, including population, devices, and outcome measures, is presented in Table 1.

The psychometric properties examined varied across the included studies, but all focused on the evaluation of functional biomechanical measures of the foot and ankle using wearable sensor technologies. Each study investigated different combinations of measurement characteristics—such as agreement with reference systems, reliability indices, and observer consistency—according to the specific aims and contexts of the analyses [14,15,16,17]. Validity was most frequently addressed through comparisons with laboratory-based, instrumented, or observational reference standards, whereas reliability-related outcomes were reported less consistently across studies. Measures of measurement error, including the standard error of measurement, were reported in a limited number of studies, while minimal detectable change and minimal clinically important difference were not reported. An overview of the study populations, wearable devices, and functional outcome measures is provided in Table 1, while a detailed description of the psychometric characteristics and numerical results reported in each study is presented in Table 2.

### 3.2. Methodological Quality and Risk of Bias

The methodological quality of the included studies was assessed using the COSMIN Risk of Bias tool, focusing on whether each study employed an appropriate design, adequate reference standards, suitable statistical methods, and transparent reporting for the specific measurement properties evaluated. Overall, the quality varied across studies, reflecting differences in sample size, measurement conditions, and the completeness of psychometric evaluation.

Mariani et al. (2013) demonstrated consistently very good methodological quality [14]. The study employed a clear design with predefined hypotheses, included an adequate sample size with more than 3000 gait cycles, and used validated pressure insoles as a robust reference standard. Statistical analyses for assessing temporal event detection were appropriate, and procedures were reported in sufficient detail to permit replication. Although minimal clinically important difference (MCID) values were not estimated, the absence of this metric did not compromise the validity of the findings. Cudejko et al. (2023) showed adequate to very good methodological rigour [15]. The use of force plates as a reference system strengthened the validity component, and the study applied suitable statistical measures such as ICC, SEM, and Bland–Altman analysis. However, the testing environment was restricted to controlled laboratory tasks, which limits ecological generalizability. Nevertheless, the authors clearly reported methodological procedures, including the handling of missing data. Marzano-Felisatti et al. (2025) demonstrated adequate methodological quality [17]. The study benefited from strong inter- and intra-observer reliability for the video-based reference method and from high ecological validity by collecting data during official competitions. At the same time, the dependence on observer-based video analysis introduced greater potential for operator-related bias, and the absence of test–retest reliability and measurement-error evaluation reduced the completeness of the psychometric assessment. Mach et al. (2025) exhibited the lowest overall methodological quality, receiving an inadequate rating [16]. Major limitations included the small sample size and the reliance on another inertial measurement unit rather than a gold-standard reference, reducing confidence in the criterion validity of the findings. Although statistical analyses were appropriate and transparently reported, the design constraints produced a high risk of bias and limited generalizability.

Across all included studies, reporting of measurement error indices (such as SEM and MDC) remained limited, and none provided MCID values. Furthermore, test–retest reliability was inconsistently assessed, and heterogeneity in reference standards and measurement conditions restricted comparability across studies. Full COSMIN ratings and item-level evaluations for each study are presented in Table 3.

### 3.3. Synthesis of Results

#### 3.3.1. Functional Biomechanical Outcome Measures of the Foot and Ankle

Across the included studies, functional biomechanical assessment primarily targeted temporal, kinetic, and task-dependent movement parameters of the foot and ankle. In gait analysis, stance phase and its constituent subphases—loading response, foot-flat, and push-off—were quantified during overground walking in healthy individuals as well as in patients with ankle osteoarthritis and post-surgical ankle conditions [14]. Laboratory-based assessments extended the scope of outcome measures to include vertical ground reaction forces and centre-of-pressure trajectories during a range of functional activities, including walking, squatting, jumping, sit-to-stand transitions, and stair ambulation [15]. In assessments involving higher mechanical demands, outcome measures were adapted to the specific movement task. Repetitive jump counts during official beach volleyball matches were used to characterize the mechanical exposure associated with take-off and landing actions [17], while running-related analyses focused on temporospatial parameters, specifically step rate and stride length, during treadmill running [16].

#### 3.3.2. Compatibility of Functional Tests with Wearable Sensor Technologies

All included studies demonstrated the feasibility of integrating wearable sensor technologies with functional biomechanical assessments of the foot and ankle, although considerable heterogeneity was observed in sensor type, placement, and testing environment. Foot-worn inertial measurement units were incorporated into overground walking assessments, allowing the extraction of stance and inner-stance phase parameters without dependence on laboratory-based motion capture systems [14]. Wireless pressure insoles enabled the acquisition of kinetic and centre-of-pressure data across a range of functional activities, including walking, squatting, jumping, sit-to-stand transitions, and stair ambulation, extending measurement beyond tasks typically restricted to force-plate instrumentation [15]. In assessments involving higher-intensity or repeated movement tasks, wearable inertial sensors were applied under both controlled and ecologically valid conditions. Trunk-mounted inertial sensors facilitated continuous monitoring of jump activity during official beach volleyball matches without interfering with task execution [17], while foot-mounted inertial sensors yielded temporospatial running parameters comparable to those obtained from shank-mounted systems during treadmill running [16].

#### 3.3.3. Psychometric Properties of Wearable-Based Biomechanical Assessments

Across the included studies, psychometric evaluation predominantly addressed agreement with reference standards, with more limited and variable reporting of reliability-related outcomes. Temporal gait parameters derived from foot-worn inertial measurement units demonstrated close time-based agreement with plantar pressure insoles, with small mean differences observed across stance and inner-stance phases, indicating accurate temporal characterization of gait events using wearable sensors [14]. Wireless pressure insoles exhibited fair to excellent test–retest reliability for kinetic and centre-of-pressure outcomes across multiple functional activities, in addition to acceptable agreement with force plate measurements [15]. Agreement-based validation was also reported for task-specific movement assessments. Inertial sensor-based jump detection demonstrated high sensitivity when compared with video-based observational analysis during repeated jumping tasks performed under competitive conditions [17]. Similarly, foot-placed inertial sensors showed strong associations with shank-mounted inertial sensors for temporospatial running parameters, including step rate and stride length, supporting consistency between sensor placements for these outcomes [16]. Despite these findings, reporting of measurement error indices was inconsistent across studies, and none of the included investigations reported minimal detectable change or minimal clinically important difference values, limiting interpretation of the clinical or functional relevance of observed measurement differences.

## 4. Discussion

This systematic review identified four studies that applied wearable technologies to functional biomechanical tests of the foot and ankle across clinical, laboratory, and competition settings. Collectively, the evidence shows that (i) foot-worn IMUs can recover stance and inner-stance temporal events during overground walking with small mean differences relative to in-shoe pressure reference systems; (ii) wireless pressure insoles provide fair-to-excellent test–retest reliability and acceptable agreement with force plates for vertical GRF and centre-of-pressure trajectories across multiple tasks; (iii) trunk/foot-mounted IMUs can quantify high-demand sport actions (e.g., jump counts in official matches) with high sensitivity; and (iv) sensor placement (foot vs. shank) yields comparable temporospatial running metrics for step rate and stride length under treadmill conditions. However, reliability indices and measurement error (SEM/MDC) were inconsistently reported, and no study reported MCID, limiting interpretability for clinical decision-making.

### 4.1. Relationship to Prior Work

Our synthesis aligns with prior reviews and validation studies showing that IMUs offer good-to-excellent validity and reliability for mean spatiotemporal gait parameters (e.g., step/stride time and length), whereas metrics derived from variability or symmetry are less stable and require stricter protocols (e.g., longer recording windows, more strides) [18,19]. Consistent with these trends, Mariani et al. (2013) demonstrated millisecond-level time agreement for stance sub-phases when foot-worn IMUs were referenced to plantar pressure insoles, supporting IMU use for clinically relevant inner-stance segmentation [14]. For kinetics, our findings echo contemporary work indicating that pressure insoles underestimate vertical GRF versus force plates but yield acceptable agreement and reliability for task-level comparisons [15,20]. In running, meta-analytic evidence reports excellent concurrent validity for step frequency/stride length and good reliability for stance time with IMUs [21], which is coherent with Mach et al. (2025) showing strong agreement between foot- and shank-mounted sensors for these variables [16]. Finally, sport-specific jump monitoring using body-mounted IMUs has matured to high sensitivity in official competitions [17], reinforcing feasibility in valid environments.

### 4.2. Psychometric and Methodological Considerations

A central observation is the imbalance between validity testing and complete psychometric characterization. Only a subset of studies reported test–retest reliability and SEM, and none reported MDC/MCID, precluding inferences about real change versus noise in clinical follow-up. This gap contrasts with best practice in measurement science, where SEM and MDC are recommended to distinguish true change from error [22], and MCID is advocated to link change scores with patient relevance [23]. For gait assessment in foot/ankle pathology and post-surgical states, foot-worn IMUs can recover stance-phase sub-events and durations with precision sufficient for temporal profiling (e.g., prolonged loading response or reduced push-off), potentially aiding progress monitoring and return-to-activity decisions [14,18]. Pressure insoles provide a portable kinetic proxy to characterize vGRF and COP outside the lab; although their absolute values can deviate from force plates, within-patient tracking under consistent conditions is promising for clinics and training environments [15,20]. In sport, competition-grade jump detection and running temporospatial metrics are sufficiently mature for external-load monitoring and technique interventions, provided that device- and context-specific limitations are acknowledged [16,17,21].

### 4.3. Public Health Implications and Global Perspectives

Foot and ankle disorders constitute a substantial public-health burden due to their high incidence, recurrence rates, and long-term consequences for mobility, occupational participation, and quality of life [1,3,4]. The findings of this review indicate that wearable sensor-based functional biomechanical assessments may support public-health strategies aimed at early detection of movement deficits, load-management monitoring, and secondary prevention of injury recurrence. Compared with traditional laboratory-based biomechanics, wearable technologies offer greater portability, scalability, and feasibility for repeated assessment across clinical, community, and sport settings [8,18,19].

Differences in healthcare infrastructure and economic development across continents substantially influence the adoption and applied value of wearable biomechanical technologies. In high-income regions, such as Europe and North America, wearable sensors are primarily used to complement laboratory-based gait analysis and force-plate assessments, enabling longitudinal monitoring beyond the clinic and supporting data-informed rehabilitation and return-to-sport decisions [18,21]. In these contexts, the main challenge lies not in technological access but in the lack of standardized protocols and incomplete reporting of psychometric properties, particularly measurement error and clinically meaningful change, which limits clinical interpretability and broader public-health application [22,23]. In low- and middle-income regions, where access to motion-capture laboratories, force plates, and specialized personnel is often restricted, wearable sensors may function as a primary biomechanical assessment tool rather than an adjunct. Prior work on wearable gait analysis highlights their potential to decentralize biomechanical assessment and extend care into community-based, occupational, and athletic environments [8,18]. However, the external validity of wearable-derived metrics across diverse populations, footwear habits, and environmental conditions remains insufficiently studied, underscoring the need for region-specific validation and reporting of psychometric quality [19,21].

Across all healthcare systems, the absence of consistently reported standard error of measurement (SEM), minimal detectable change (MDC), and minimal clinically important difference (MCID) values also observed in the studies included in this review represents a critical limitation for translating wearable biomechanics into public-health decision-making. These parameters are essential to distinguish true functional change from normal measurement variability and to support large-scale screening, monitoring, and evaluation of intervention effectiveness [22,23].

### 4.4. Opportunities for AI and Digital Health Integration

Although none of the included studies explicitly incorporated AI-based methods or comprehensive digital health frameworks, several methodological features indicate potential avenues for future integration. All studies involved the acquisition of high-resolution, continuous biomechanical data during walking, functional tasks, running, or repetitive movement activities, thereby generating datasets suitable for advanced analytical approaches [14,15,16,17]. The demonstrated compatibility of wearable sensors with both controlled and real-world assessment environments further supports the feasibility of extended monitoring beyond laboratory settings. These characteristics provide a basis for the future application of data-driven techniques, including machine learning, to improve event detection, movement pattern classification, and automated outcome extraction. In addition, integration within digital health infrastructures may enable longitudinal assessment, individualized feedback, and decision-support tools to assist clinicians and practitioners in rehabilitation and performance-related contexts.

### 4.5. Strengths and Limitations of This Review

Strengths include a focused lens on functional tests compatible with wearable sensors. However, the evidence base was small and heterogeneous (populations, tasks, devices, references), precluding meta-analysis and limiting generalizability. Our search strategy did not include specific terms to identify experimental study designs, which may have led to the omission of relevant experimental studies addressing aspects within the scope of this review. Furthermore, several included/related studies employed laboratory comparators that are not universally accepted as gold standards for all outcomes (e.g., using another IMU as a criterion), which warrants caution in interpreting criterion validity (e.g., Mach et al., 2025) [16]. Finally, inconsistent reporting of measurement error and responsiveness constrained our ability to translate effect sizes into clinically meaningful thresholds [23].

### 4.6. Future Directions

Future research should prioritize large-scale, multicentre studies that include diverse populations across continents and healthcare systems. Such studies should adopt harmonized protocols and explicitly report validity, test–retest reliability, SEM, MDC, and, where feasible, MCID to enhance clinical and public-health interpretability. From a global health perspective, validation under resource-limited conditions and in community-based settings is particularly important to support equitable implementation.

Integration of wearable technologies into digital health ecosystems represents a promising avenue for scaling biomechanical assessment. Longitudinal monitoring, combined with cloud-based data storage and automated analysis, could facilitate population-level surveillance of mobility impairments and injury risk. In this context, artificial intelligence and machine-learning methods may enable automated event detection, pattern recognition, and individualized risk stratification. However, transparent development and validation of such models, following reporting frameworks such as TRIPOD-AI [24], will be essential to ensure clinical trust and reproducibility.

Ultimately, aligning wearable biomechanical assessment with public-health objectives requires not only technical validation but also implementation research that addresses clinician training, data governance, patient engagement, and cost-effectiveness across different healthcare systems.

## 5. Conclusions

The available evidence suggests that wearable technologies, such as IMUs and pressure insoles, show potential for supporting functional biomechanical assessment of the foot and ankle, particularly in capturing temporospatial parameters and task-specific outcomes across clinical and sport contexts. However, this evidence is currently limited to a small number of heterogeneous studies with modest sample sizes and incomplete psychometric evaluation. In particular, the inconsistent reporting of reliability, measurement error (SEM/MDC), and the absence of MCID values restrict the interpretation of these tools for clinical decision-making. Therefore, current findings should be interpreted with caution, and definitive conclusions regarding their validity and reliability cannot yet be established.

## Figures and Tables

**Table 1:** Summary of included studies evaluating functional biomechanical assessment of the foot and ankle.

Author (Year)	Study Title	Population	Device	Outcome Measure
Mariani et al., 2013 [14]	Quantitative estimation of the foot-flat and stance phase of gait using foot-worn inertial sensors	42 participants were included: 10 healthy controls 12 ankle osteoarthritis 11 post-total ankle replacement 9 post-ankle arthrodesis	Foot-worn inertial measurement unit (IMU) 3D accelerometers 3D gyroscopes Sensor placed on the forefoot Sampling frequency: 200 Hz Reference system: validated plantar pressure insoles (Pedar-X) used as the gold standard for temporal event detection	Overground walking test; Two trials of 50 m walking at self-selected speed; Functional outcomes derived from temporal gait parameters, specifically: Stance phase Loading response Foot-flat Push-off
Cudejko et al., 2023 [15]	Wireless pressure insoles for measuring ground reaction forces and trajectories of the centre of pressure during functional activities	Healthy adults (*n* = 21; age 30.8 ± 9.0 years; BMI 25.2 ± 3.9; 67% male, 33% female)	X4 Foot and Gait Measurement System^®^ (XSENSOR wireless pressure insoles)	Squats, vertical jumps, 30-s sit-to-stand, walking, stair ascent/descent (vGRF, peak pressure, and COP ML and AP trajectories)
Mach et al., 2025 [16]	Foot-Placed Inertial Measurement Units Are Valid Against Shank- Placed Units When Measuring Temporospatial Running Variables	Nine adults (5 males, 4 females; mean age 28.33 ± 5.78 years; height 1.75 ± 0.11 m; mass 74.06 ± 16.24 kg), injury-free runners.	Foot-placed: RunScribe IMUs Shank-placed (criterion): MyoMotion IMUs (Noraxon USA)	Running temporospatial variables on a treadmill: Step rate (SR) Stride length (SL)
Marzano-Felisatti et al., 2025 [17]	Validation of the WIMU PROTM Device for Jump Detection in Beach Volleyball: A Gender-Based Analysis during Official Competitions	Eleven beach volleyball players (6 female and 5 male)	high-definition cameras and the WIMU PROTM device. WIMU PRO™ inertial measurement unit (RealTrack Systems, Almeria, Spain).	Jump detection during official beach volleyball matches (serve, spike, block, others).

**Table 2:** Psychometric characteristics and reported results of the included studies.

Study	Test–Retest Reliability	Intra-Rater Reliability	Inter-Rater Reliability	Validity	SEM	MDC	MCID
Mariani et al., 2013 [14]	Not assessed	Not assessed	Not assessed	Time-based agreement between IMU-derived and pressure-insole-derived gait events; mean differences 1–19 ms across stance phases	Not reported	Not reported	Not reported
Cudejko et al., 2023 [15]	Repeated-session measurements within one day; ICCs ~0.55–0.95 depending on task and variable	Not assessed	Not assessed	Comparison of wireless pressure insoles with force plates for vGRF and COP trajectories (Bland–Altman agreement)	Reported for vGRF and COP outcomes (task-dependent)	Not reported	Not reported
Mach et al., 2025 [16]	Not assessed	Not assessed	Not assessed	Agreement between foot- and shank-placed IMUs; step rate r = 0.90 (ICC = 0.91), stride length r = 0.80 (ICC = 0.80)	Not reported	Not reported	Not reported
Marzano-Felisatti et al., 2025 [17]	Not assessed	Intra-observer agreement κ = 0.99 (video analysis)	Inter-observer agreement κ = 0.99 (video analysis)	Jump-by-jump agreement between IMU and video observation; overall sensitivity 96.29%	Not reported	Not reported	Not reported

**Table 3:** COSMIN methodological quality and risk-of-bias assessment for included studies.

Study	Study Design	Sample Size	Reference Standard	Measurement Conditions	Reliability	Measurement Error	Statistical Methods	Missing Data	Blinding	Transparency/Reproducibility	Overall COSMIN Judgement
Mariani et al., 2013 [14]	Very good (clear design; predefined hypothesis)	Very good (42 participants; >3000 gait cycles)	Very good (pressure insoles)	Very good (inertial sensors and pressure insoles)	N/A	Adequate (MCID was not calculated)	Very good	Adequate (no formal imputation)	N/A	Very good (allows replication)	Adequate
Cudejko et al., 2023 [15]	Adequate (lab-based validation)	Adequate (*n* = 21; justified)	Very good (force plates)	Adequate (lab tasks only)	Very good	Adequate	Appropriate (ICC, SEM, BA)	Clearly reported	Clearly reported	Adequate (reproducibility limited)	Very good
Mach et al., 2025 [16]	Adequate (IMUs)	Doubtful/Inadequate (*n* = 9)	Doubtful (not gold standard)	Adequate (controlled treadmill protocol)	N/A	N/A	Very good (ICCs and vland-Altman)	Adequate (no missing data)	N/A	Adequate (algorithms were device-dependent)	Inadequate (high risk of bias, due to small sample size and lack of a gold standard reference.)
Marzano-Felisatti et al., 2025 [17]	Adequare (external reference)	Very good (1481 jumps)	Adequate (video-based is operator-dependent)	Adequate (sport-specific conditions)	N/A (wearable devices)	N/A	Appropriate (sensitivity, FP/FN))	Adequate. Observer reliability reported	N/A	Adequate (reproducibility limited)	Adequate

## Data Availability

No new data were created or analyzed in this study.

## Supplementary Materials

The following supporting information can be downloaded at https://www.mdpi.com/article/10.3390/jcm15103892/s1. PRISMA 2020 Checklist.

## Appendix A

### Search Strategy Used in All Databases

(“Wearable Electronic Devices” OR “Wearable” OR “inertial measurement unit” OR “IMU” OR “accelerometer” OR “gyroscope” OR “magnetometer” OR “pressure insoles” OR “instrumented insoles” OR “force-sensitive resistors” OR “FSR” OR “plantar pressure sensors” OR “smart socks” OR “smart insoles” OR “smart footwear” OR “textile sensors” OR “wireless sensors” OR “capacity sensors” OR “piezoresistive” OR “piezoelectric”) AND (“Psychometrics” OR “Psychometric properties” OR “Reliability” OR “Test–retest reliability” OR “Intraexaminer reliability” OR “Intrarater reliability” OR “Interrater reliability” OR “Interrater reliability” OR “Validity” OR “Convergent validity” OR “Discriminant validity” OR “Concurrent validity” OR “Predictive validity” OR “Responsiveness” OR “Minimal Detectable Change” OR “MDC” OR “Minimal Important Difference” OR “MID” OR “Minimal Clinically Important Difference” OR “MCID”) AND (“Observational” OR “cohort studies” OR “case–control” OR “cross-sectional studies”) AND (“ankle” OR “foot” OR “feet” OR “foot biomechanics”)
